# Risk Factors Associated With Low-Birth-Weight Babies in Tertiary Health Care Facilities in Lahore, Pakistan: A Case-Control Study

**DOI:** 10.7759/cureus.63832

**Published:** 2024-07-04

**Authors:** Naila Bajwa, Meha Siddiqui, Muhammad Awais, Alishba Rasool, Ayesha Amin, Mahnoor Khan, Saira Afzal

**Affiliations:** 1 Community Medicine, King Edward Medical University, Lahore, PAK; 2 Community Medicine, Institute of Public Health, Lahore, PAK; 3 Public Health and Preventive Medicine, King Edward Medical University, Lahore, PAK

**Keywords:** low-birth-weight babies, nutritional status, parity, anemia, maternal risk factors

## Abstract

Introduction

Low birth weight (LBW) is a well-known contributing factor to neonatal health, emphasizing the importance of maternal health and socio-economic conditions. The birth weight of a newborn is a major public health problem, which is more common in low-middle-income countries (LMICs).

Objective

The objective of this study is to assess the association of different socio-economic and maternal factors with LBW babies in Lahore.

Methods

This case-control study was carried out at the Obstetrics and Gynecological Department in Mayo Hospital, Lahore, Pakistan from September 25, 2023 to December 31, 2023. A total of 186 mothers who delivered in the maternity ward, categorized into two groups (93 cases and 93 controls), were included and data was collected with the help of a self-administered structured tool. A chi-square test was used to identify maternal risk factors significant for LBW babies. The strength of association between maternal risk factors and LBW babies was presented using the odds ratio (OR) with the respective 95% confidence interval (CI).

Results

The study revealed that maternal anemia [OR: 3.378, 95% CI: 1.568, 7.275] and inadequate nutritional status [OR: 1.031, 95% CI: 0.014, 0.071] were more likely to cause delivery of LBW babies. Regarding socio-demographic factors, household income < 25000 [OR: 5.185, 95% CI: 2.770, 9.707] and illiterate mothers [OR: 3.325, 95% CI: 1.820, 6.074] were associated with increased likelihood of LBW babies. Maternal age < 20 had a strong association [OR: 10.920, 95% CI: 2.455,48.575] with delivery of LBW children.

Conclusion

The study concludes that multiple risk factors including anemia, inadequate nutritional status, household income < 25000, illiterate mother, and maternal age < 20 are strongly associated with LBW babies. It is apparent that a multimodal strategy is necessary to reduce the risk of LBW babies.

## Introduction

Low birth weight (LBW) is a crucial indicator of intrauterine growth and the single most important factor influencing neonatal survival. It is also a critical indicator of infant mortality and one of the easiest-to-understand and most comprehensive health indices in each community [1]. According to the World Health Organization, infants weighing less than 2500 grams are classified as LBW babies [1]. The mortality rate for infants under 2500 g is 40 times higher than that of infants over 1500 g, and the mortality rate for infants under 1500 g is 200 times higher than that of infants over 2500 g [1].

The birth weight of a newborn is a major public health problem, which affects an estimated 20 million babies born annually and is more common in low-middle-income countries (LMICs) [2]. Of these, 28% are born in South Africa, 13% in Sub-Saharan Africa, and 9% in Latin America [2]. A recent study by Sathi et al. showed that, in comparison to other LMICs in South Asia, the prevalence of LBW recorded in Afghanistan, Bangladesh, Nepal, and Pakistan was 15.13%, 14.93%, 11.73%, and 19.18%, respectively [3]. LBW is assumed to be caused by intrauterine growth retardation (IUGR) and preterm delivery; the etiology of LBW is uncertain [4]. IUGR is caused by insufficient uterine-placental perfusion and poor fetal nutrition, which affect the fetus's overall anthropometric characteristics [4].

LBW is a challenging global public health problem because it has many complications, like eye problems, deafness, neurological problems, developmental delays, low IQ, and behavioral disorders [5]. In West Ethiopia, a study revealed the prevalence of risk factors among cases and controls, like maternal age less than 20 being reported in 9.7% of cases and 17.2% of controls, inadequate food intake being reported in 93.5% of cases and 62.9% of controls, and informal education of mothers reported in 34.4% cases and 17.2% controls [6]. According to a study also conducted in Ethiopia, the reported risk factor among cases and controls was no ANC visits (14.2% and 10.2%), respectively. The second risk factor identified was a short pregnancy interval < 24 months (22% in cases and 22.2% in controls) [7].

In Pakistan, one study was conducted in the Malakand division, and the reported risk factors were maternal anemia reported among 49.3% of cases and 47.6% of controls, and a monthly income less than 10000 PKR reported in 69.8% of cases and 74.2 percent of controls [8]. Risk factors and nutritional conditions are especially important in influencing the birth weight of a newborn. For the fetus to grow and the birth weight to be at its ideal level, there is a strong need to educate the mothers [9].

Many studies address different risk factors for mothers, like maternal age, bad obstetric history, anemia, and hypertension, which have a strong association with the LBW of neonates [6], but few studies have been conducted with regard to maternal nutritional status and dietary practices during pregnancy that contribute to LBW. So the main objective of this study is to assess the nutritional status of mothers, socio-demographic and maternal risk factors related to LBW babies delivered in tertiary health care facilities in Lahore.

## Materials and methods

A case-control study was carried out in the Obstetrics and Gynecological Department of Mayo Hospital Lahore among 186 mothers who delivered in the maternity ward. A non-probability consecutive sampling technique was used for participants in the study after obtaining ethical approval from the institutional review board. The sample size of 186 (93 cases and 93 controls) was calculated with 95% confidence interval (CI), 70% power of test, and percentage of no ANC visit of 29.4% in cases and 14.4% in controls using the Epi Info calculator [5]. Mothers who delivered live-term babies singleton with a birth weight less than 2500 g were taken as cases [10] and those with a birth weight of 2500 g or more were taken as controls [10] irrespective of the mode of delivery included in the study. Mothers having antepartum hemorrhage or UTIs, admitted to the ICU, delivered twin babies, and could not communicate due to serious medical conditions were excluded from the study. Data were collected with the help of self-administered, close-ended Proforma for gathering information from September 25, 2023 to December 31, 2023. The Proforma was adopted from similar previous studies and consists of questions on the biodata of the patient, like name, age, birth weight of the baby, parity, and the husband's occupation. Data on potential maternal risk factors like household income < 25000, no antenatal visits, inter-pregnancy interval <24 months, illiterate mothers, maternal age <20, and maternal anemia [6-8] were obtained. The food frequency questionnaire had 10 food groups. Pregnant women were assigned adequate if they consumed more than five food groups and inadequate if they consumed less than five food groups [11]. The collected data were entered and analyzed using IBM SPSS Statistics for Windows, Version 24 (Released 2015; IBM Corp., Armonk, New York, United States). For descriptive analysis mean and standard deviations were calculated while percentages and frequencies were given for categorical variables. Inferential analysis was performed using the chi-square test and the p-value (< 0.05) was considered significant. The strength of association between LBW babies and risk factors was tested by using the odds ratio (OR) and 95% CI. Selection bias was often present in case-control studies. Controls should be chosen from the same representative groups that generated the cases in order to reduce selection bias. In our study, samples were selected from the same maternity ward used for cases and controls.

## Results

This study included a total of 186 mothers who delivered in the maternity ward, and categories were divided into two groups: 93 cases and 93 controls. The mean maternal age of the study participants was 26.18 ± 4.618. The maximum age was 40 and the minimum age was 18 in this study. The mean birth weight of the babies was 2.667 ± 0.4377. The minimum weight was 1.8 kg and the maximum weight was 4 kg in this study. The mean hemoglobin of the delivered mothers was 10.73 ± 1.302. The minimum hemoglobin was 8 g/dl, and the maximum hemoglobin was 15 g/dl in this study.

Almost 67.7% of mothers in the case group and 38.7% of mothers in the control group had no formal education. A higher proportion of mothers in the cases (26.9%) had more than three children and 11.8% of fathers were unemployed among cases (Table 1). Household income < 25000 was reported in 65.6% of cases. Regarding the reproduction profile, a high proportion of no antenatal visits were reported in 63.4% of cases. Interpregnancy intervals < 24 months were reported in 72.0% of cases. The nutritional status of 79.6% of mothers in the case group was inadequate and maternal anemia was also found in 88.2% of cases (Table 2).

**Table 1:** Frequency of socio-demographic variables among cases and controls

Variable	Categories	Case (n = 93) N (%)	Control (n = 93) N (%)
Father occupation	Unemployed	11(11.8)	10(10.8)
	Employed	82(88.2)	83(89.2)
Parity	< 3 Children	68(73.1)	70 (75.3)
	>3 Children	25(26.9)	23 (24.7)
Mother education	No formal education	63(67.7)	36(38.7)
	Formal education	30(32.3)	57(61.3)

**Table 2:** Frequency of maternal risk factors among cases and controls

Maternal Risk Factors	Categories	Case (n = 93) N (%)	Control (n = 93) N (%)
Household income < 25000	No	32(34.4)	68(73.1)
	Yes	61(65.6)	25(26.9)
Interpregnancy interval < 24 months	No	26(28.0)	31(33.3)
	Yes	67(72.0)	62(66.7)
Antenatal visits	No	59(63.4)	72(77.4)
	Yes	34(36.6)	21(22.6)
Maternal age < 20	No	75(80.6)	91(97.8)
	Yes	18(19.4)	2(2.2)
Illiterate mothers	No	30(32.3)	57 (61.3)
	Yes	63(67.7)	36(38.7)
Maternal anemia	No	11(11.8)	29(31.2)
	Yes	82(88.2)	64(68.8)
Nutritional status	Inadequate	74(79.6)	10(10.8)
	Adequate	19(20.4)	83(89.2)

**Figure 1:**
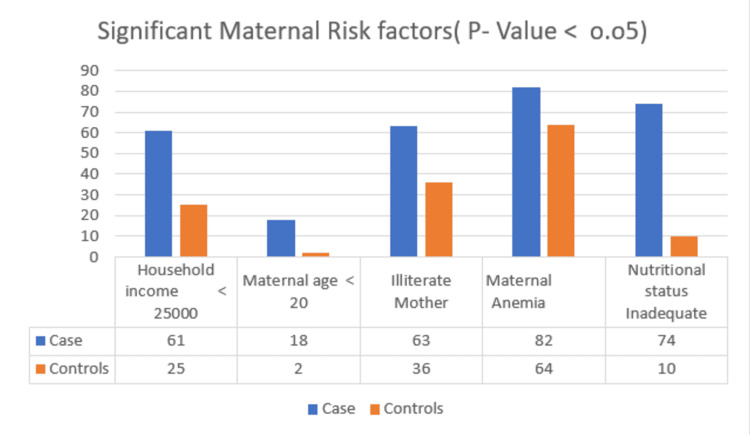
Significant maternal risk factors

In inferential analysis, a chi-square test was applied. The p-values for household income < 25000, maternal age < 20, illiterate mothers, maternal anemia, and nutritional status were statistically significant (p-value <0.05). For the interpregnancy interval and no antenatal visits, the result was not significant (p-value > 0.05) (Figure 1). The OR and CI were calculated for the strength of association, and higher ORs were reported in household income < 25000, no antenatal visits, maternal age < 20, illiterate mothers, maternal anemia, and nutritional status of mothers (Table 3).

**Table 3:** Strength of association between maternal risk factors and low-birth-weight babies

Maternal Risk Factor	Categories	Case Control			Odds Ratio (95% CI)	Chi Square (p-value)
		Case N (%)	Control N (%)			
Household income < 25000	No	32 32.0%		68 68.0%	5.185 (2.770, 9.707)	28.030 (0.000)
	Yes	61 70.9%		25 29.1%		
Interpregnancy interval <24	No	26 45.6%		31 54.4%	1.288 (0.689, 2.408)	0 .632 (0.426)
	Yes	67 51.9%		62 48.1%		
Maternal age < 20	No	75 45.2%		91 54.8%	10.920 (2.455,48.575)	14.342 (0.000)
	Yes	18 90.0%		2 10.0%		
Illiterate mothers	No	30 34.5%		57 65.5%	3.325 (1.820, 6.074)	15.743 (0.000)
	Yes	63 63.6%		36 36.4%		
Maternal anemia	No	11 27.5%		29 72.5%	3.378 (1.568, 7.275)	10.319 (0.001)
	Yes	82 56.2%		64 43.8%		
Nutritional status	Inadequate	74 88.1%		10 11.9%	1.031 (0.014, 0.071)	88.919 (0.000)
	Adequate	19 18.6%		83 81.4%		
No antenatal visits	No	59 45.0%		72 55.0%	10.920 (2.455, 48.575)	5.920 (0.052)
	Yes	34 63.0%		20 37.0%		

## Discussion

The study aims to find the association between maternal risk factors and LBW babies. The study revealed that mothers having anemia and inadequate nutritional status were more prone to deliver LBW babies. Regarding socio-demographic factors, household income < 25000 and illiterate mothers were associated with an increased likelihood of LBW babies. Maternal age < 20 had a strong association with delivering LBW children. These findings suggest that mothers of newborns with adverse social determinants continue to be at a high risk of delivering LBW babies. The results of our study were consistent with the results of a nationally representative cross-sectional survey conducted in India on the prevalence and determinants of LBW babies [12] and also, a population-based study conducted in Bangladesh [13].

The most common medical condition during pregnancy was anemia. Our study also provided the frequency of maternal anemia among cases and controls, which was 56.2% and 43.8%, respectively. The odds of an event of maternal anemia were approximately 3.3 times higher in the exposed group. Anemia has been linked to significant variations in birth weight at any point in the pregnancy, particularly during the third trimester because rapid fetal growth occurs at this stage [14].

The impact of maternal nutritional status on birth outcomes, particularly the association with LBW babies, was a crucial aspect of maternal and child health. This finding underscores the significance of adequate maternal nutrition for optimal fetal development. Our study provides significant evidence that inadequate nutritional status among cases was strongly associated with LBW babies.

Similar to our findings, a study conducted in Nepal proposed a link between poor nutritional intake and LBW. Mothers who took food less than two times had a 2 times higher chance of delivering LBW babies [15]. In another study, the nutritional status of pregnant women revealed that mothers who had experienced malnutrition during their pregnancies were more likely to give birth to underweight children [16].

The socioeconomic position of households and how income affects birth outcomes were essential to comprehending the maternal risk factors linked to LBW babies. The importance of household income < 25000 in cases of our study provides significant evidence with LBW babies. A hospital-based cross-sectional study in India provides compelling evidence for the high correlation between a lower household income and a higher chance of having LBW children. The study sheds insight into the differences in socioeconomic status that affect the health of mothers and children [17]. Identical results were also shown by a case-control study conducted in India [18].

An essential component of comprehending maternal risk factors was the impact of maternal age on birth outcomes, specifically the correlation between being under 20 years old and the incidence of LBW. The significance of young maternal age on birth weight outcomes in both cases and controls was highlighted by our study, which shows the frequency of LBW in maternal age < 20 years was 90.0% and 10.0%, respectively. In a study carried out in the Slovak Republic, there was a higher risk of LBW at maternal ages less than or equivalent to 18 years [19]. Similarly, numerous other researchers suggest that LBW may be connected with maternal ages older than 34 and less than or equivalent to 18 years old [20,21]. Consideration of methodological aspects, such as the definition of young maternal age, measurement tools, and diagnostic criteria, was essential for a meaningful comparison across studies.

The impact of maternal education on birth outcomes, particularly the association between illiteracy and the occurrence of LBW, was a critical aspect of maternal risk factors. Our finding supports the strong evidence of maternal illiteracy in the cases was 67.7% in the context of delivering LBW babies. One study shows a strong correlation between maternal illiteracy and the likelihood of having a LBW baby. An Indian study conducted that shows low birth outcomes were significantly correlated with mothers' education. Illiterate mothers frequently lack optimum antenatal and nutrition, which stunts fetal growth and increases the risk of LBW and preterm birth in babies. These findings provide a body of evidence supporting the impact of maternal illiteracy on LBW [22].

The following methodological flaws should be taken into account when interpreting the study's results: Recall errors in the measurement of exposures (such as the frequency of consumption of various food groups or events associated with household food insecurity) are conceivable because the study used a retrospective approach. These mistakes may result in exposures being incorrectly classified, which could ultimately lead to an underestimation of the strength of correlations. Furthermore, residual confounding from unmeasured or incorrectly categorized factors cannot be completely eliminated in observational studies. Furthermore, we did not examine several potentially predictive factors of LBW, such as pre-pregnancy weight, gestational weight increase, and paternal anthropometry, because the study was case-control in nature. These findings would not be generalized because the study was confined to the health institution of one district.

The study is helpful for the present ongoing community and institution-based interventions to address the concerns based on the evidence for causing LBW, particularly for planners and managers should be taken into consideration when planning to enhance maternal health and lower the risk of LBW.

## Conclusions

A multimodal strategy is required to lower the risk of LBW newborns, as evidenced by a strong association of various risk factors, including maternal age, anemia, nutritional status, household income, and mothers' education. By addressing these risk factors collectively, a complete approach to mother and child health can be promoted. This enables public health interventions to be tailored to specific high-risk populations. Further research and ongoing surveillance are crucial to advancing methods and ensuring that interventions remain effective and adaptable to the evolving landscape of maternal risk factors.
